# Integrating multi-omics, network pharmacology, and experimental validation to unveil the molecular mechanisms of *Fructus Xanthii* in treating asthma

**DOI:** 10.3389/fphar.2025.1722150

**Published:** 2025-12-01

**Authors:** Yiqun Dong, Zhaosen Fan, Xiaoxiao Li, Yucheng Ming, Dong Wang, Yuanda Song, Youpeng Fan

**Affiliations:** 1 Qilu Medical University, Zibo, Shandong, China; 2 Shandong Benon Biological Technology Co., Ltd., Jinan, Shandong, China; 3 Shandong Engineering Research Center of Precision Nutrition and Healthy Aging, Qilu Medical University, Zibo, Shandong, China; 4 Research Institute of Natural Products and Health Industry Innovation, Qilu Medical University, Zibo, Shandong, China

**Keywords:** *Fructus Xanthii*, asthma, network pharmacology, machine learning, molecular docking, immune infiltration

## Abstract

**Introduction:**

This study employs an integrated approach combining multi-omics analysis, network pharmacology, machine learning, and experimental validation to elucidate the molecular mechanisms of *Fructus Xanthii* (Chinese name: Cang-Er-Zi) in treating asthma.

**Methods:**

Asthma-related differentially expressed genes (DEGs) were identified from GEO datasets (GSE63142 and GSE14787). Weighted gene co-expression network analysis (WGCNA) was performed on GSE14787. Active ingredient targets of *Fructus Xanthii* were predicted using TCMSP and SwissTargetPrediction. Integration included machine learning (RF, SVM, XGB), PPI network analysis, GO/KEGG enrichment, immune infiltration (CIBERSORT), molecular docking, molecular dynamics simulations, and *in vivo* validation in an ovalbumin-induced murine asthma model.

**Results:**

Yielded 3,755 DEGs and the MEblack module correlated with asthma. Identified 1,317 potential targets, with 100 intersecting DEGs. Hub targets included HSP90AB1, CCNB1, CASP9, CDK6, NR3C1, ERBB2, and CCK. Strong binding affinities (e.g., carboxyatractyloside with HSP90AB1 at −10.09 kcal/mol) and stable complexes were confirmed. Immune profiling showed altered cell populations. *In vivo*, *Fructus Xanthii* extract reduced inflammation, cytokines (TNF-α, IL-6, IL-1β, IL-5), and hub gene expression.

**Discussion:**

*Fructus Xanthii* exerts anti-asthmatic effects by modulating HSP90AB1/IL6/TNF and PI3K-AKT pathways, regulating inflammation, cell cycle, apoptosis, and immune homeostasis, providing empirical support for multi-target traditional Chinese medicine strategies in asthma management.

## 1 Introduction

Asthma, a chronic respiratory condition characterized by airway hyperresponsiveness, inflammation, excessive mucus production, and structural alterations, commonly results in recurrent episodes and compromised pulmonary function (Porsbjerg et al., 2023). While conventional therapies such as inhaled corticosteroids and beta2-agonists have significantly improved disease control, their limitations, including side effects and incomplete symptom resolution, have spurred interest in alternative therapeutic strategies (Busse, 2019). Traditional Chinese Medicine (TCM) has emerged as a promising complementary approach due to its holistic perspective on health and its emphasis on restoring systemic balance through multi-component herbal formulations (Chan and Ng, 2020). In TCM philosophy, asthma is conceptualized as a manifestation of vital energy (Qi) imbalance, particularly involving deficiencies within the lung, spleen, and kidney systems, compounded by interference from pathogenic factors such as wind or heat (Zhou et al., 2019). Historically, TCM therapeutic interventions have been developed according to the principles of “emperor, minister, assistant, and messenger” herbs, which function synergistically to reestablish homeostatic balance and facilitate effective Qi circulation (Liu et al., 2021).

*Fructus Xanthii*, a herb traditionally utilized in Chinese medicine, has long been applied in treatments targeting diverse inflammatory and allergic conditions. In traditional Chinese medicine (TCM) practice, this botanical agent is highly regarded for its “wind-dispelling” properties and efficacy in treating exterior patterns, demonstrating therapeutic benefits for rhinosinusitis-related symptoms, cephalgia, and select musculoskeletal discomfort (Luo et al., 2020; Luo et al., 2021; Wang et al., 2023). While systematic clinical trials specifically addressing asthma remain sparse, its integration within multi-herb respiratory formulas implies a synergistic mechanism in mitigating airway inflammatory responses and alleviating associated symptoms through combined pharmacological actions (Barnes et al., 2019; Finotto, 2019; Zinellu et al., 2019).

*Fructus Xanthii* exhibits a complex array of phytochemicals, with sesquiterpene lactones serving as its principal active components. Studies on related Xanthium species have identified key bioactive molecules, including xanthinosin, xanthatin, and structurally diverse mogolides (including Mogolide A, D, E), which are isolated through advanced chromatographic techniques. These compounds are implicated in mediating the anti-inflammatory mechanisms observed in preclinical evaluations. In addition to sesquiterpene lactones, *Fructus Xanthii* exhibits a complex phytochemical profile comprising phenylpropanoids, lignans, coumarin derivatives, and multifarious flavonoids, collectively contributing to its multifaceted therapeutic properties (Han et al., 2023). Mechanistically, the pharmacological effects of *Fructus Xanthii* derive predominantly from its ability to modulate key inflammatory signaling pathways. Notably, the sesquiterpene lactones have been associated with inhibition of nuclear factor-kappa B (NF-κB) nuclear translocation and modulation of mitogen-activated protein kinase (MAPK) signaling cascades. This molecular intervention effectively suppresses downstream transcriptional activation, thereby curtailing synthesis and secretion of pro-inflammatory mediators, including cytokines and chemokines. Consequently, the therapeutic agents mitigate pathological recruitment of immune cells to inflamed tissue microenvironments, establishing a critical mechanism for their anti-inflammatory effects (Han et al., 2023). The ability of *Fructus Xanthii* to modulate inflammatory mediators positions it as a promising therapeutic candidate for chronic inflammatory disorders typified by asthma, particularly given its impact on hallmark pathological features such as airway hyperresponsiveness and hypersecretion of mucins (Athari, 2019; Peters et al., 2019). Notably, its proposed immunomodulatory activity involves recalibrating Th1/Th2 immune responses (Han et al., 2022). Despite these insights, comprehensive elucidation of the molecular pathways linking *Fructus Xanthii*’s bioactive constituents to asthma pathophysiology remains an unmet research objective.

In the present study, we employed an integrated multi-omics approach, encompassing differential gene expression analysis from GEO datasets (GSE63142 and GSE14787) and weighted gene co-expression network analysis (WGCNA), to identify asthma-related differentially expressed genes (DEGs) and core modules. This was complemented by a network pharmacology framework to predict active ingredients and targets of *Fructus Xanthii*, constructing a “disease-drug-ingredient-target” network and performing protein-protein interaction (PPI) analysis. Pivotal therapeutic targets were systematically mapped through the integration of machine learning algorithms (random forest, support vector machine, and extreme gradient boosting) with PPI network topology. Computational validation was achieved via Gene Ontology (GO) and Kyoto Encyclopedia of Genes and Genomes (KEGG) enrichment analyses, immune cell infiltration profiling using CIBERSORT and ESTIMATE algorithms, molecular docking, and molecular dynamics simulations, confirming stable binding affinities and complex dynamics for key compounds such as carboxyatractyloside with hub targets (e.g., HSP90AB1, CCNB1, and CASP9). These *in silico* findings were substantiated by *in vivo* experimental validation in an ovalbumin-induced murine asthma model, demonstrating dose-dependent anti-inflammatory effects of *Fructus Xanthii* aqueous extract, including reduced cytokine levels (TNF-α, IL-6, IL-1β, IL-5), ameliorated lung histopathology, and downregulated expression of hub genes (HSP90AB1, CCNB1, CASP9, PI3K, AKT1). Collectively, our results elucidate the multi-target anti-asthmatic mechanisms of *Fructus Xanthii*, involving modulation of inflammation, cell cycle progression, apoptosis, and immune balance through pathways such as HSP90AB1/IL6/TNF and PI3K-AKT, thereby providing robust scientific evidence for the application of traditional Chinese medicine in asthma management.

## 2 Materials and methods

### 2.1 Prediction of active ingredients and targets of *Fructus Xanthii*

This study employed a multi-platform integrated analysis strategy to dissect the pharmacological mechanisms of *Fructus Xanthii*. Initially, chemical constituents of the herb were extracted through the Traditional Chinese Medicine Systems Pharmacology Database and Analysis Platform (TCMSP, https://www.tcmsp-e.com/). We then identified qualified active ingredients based on pharmacokinetic (ADME) parameters, retaining molecules with oral bioavailability (OB) ≥30% and drug-likeness (DL) ≥0.18. Subsequently, SMILES structural formulas of the target components were acquired from the PubChem database (https://pubchem.ncbi.nlm.nih.gov/) and imported into the SwissTarget Prediction platform (https://www.swisstargetprediction.ch/) for virtual screening of potential therapeutic targets. The target gene names were then standardized through the UniProt database (https://www.uniprot.org/) (Xing et al., 2018).

### 2.2 Collection of asthma dataset

We retrieved asthma-related gene expression datasets from the Gene Expression Omnibus (GEO) database using the keywords “asthma” and “*Homo sapiens*”. For differential gene analysis, we selected GSE63142 (27 healthy controls vs. 56 severe asthma samples) and GSE14787 (13 healthy controls vs. 60 severe asthma samples). After filtering, 27 normal and 56 severe asthma samples from GSE63142 were used for DEG identification. The GSE14787 dataset was integrated to construct immune infiltration and weighted gene co-expression networks (WGCNA) (Zhou et al., 2025).

### 2.3 Building a weighted Co-expression network

We further processed the GSE14787 dataset through weighted gene co-expression network analysis (WGCNA) using R version 4.1.2 to identify co-expressed gene modules. Initially, we performed hierarchical clustering on the research samples to detect and eliminate outlier samples. Subsequently, we constructed a scale-free network and utilized the pick Soft Threshold function to select an appropriate soft threshold. Following this, we established an adjacency matrix and transformed it into a topological overlap matrix (TOM), employing various degrees to generate a gene tree diagram with module colors. Finally, we calculated the correlation between each module and the differential samples (Liu et al., 2025).

### 2.4 Identification of differentially expressed genes in asthma

Subsequently, we standardized the GSE63142 dataset using the “Sva” package in R 4.1.2. Differentially expressed genes (DEGs) were identified using the “limma” package with selection criteria of absolute log2 fold change >1 and adjusted P-value <0.05. Heatmaps and volcano plots visualizing the DEGs were generated using the “pheatmap” and “ggplot2” packages in R 4.1.2 (Feng et al., 2025).

### 2.5 Intersection target acquisition of *Fructus Xanthii* for asthma treatment

We utilized the SRplot (https://www.bioinformatics.com.cn/) to generate Venn diagrams. The identified asthma-related genes, target genes, and active ingredients of *Fructus Xanthii* were imported into Cytoscape 3.8.1 for visual data analysis to construct a “disease-drug-active ingredient-target” network. The overlapping target genes identified through this analysis were predicted as potential therapeutic targets of *Fructus Xanthii* in asthma treatment (Guo et al., 2025).

### 2.6 Construction of protein interaction network

The intersected target dataset was uploaded to the STRING database (https://string-db.org/) with *Homo sapiens* as the organism and an interaction score threshold ≥0.4 to construct a refined protein-protein interaction (PPI) network. Network visualization and functional analysis were performed using Cytoscape v3.10.2. Key analyses included.

1. Topological profiling via CytoNCA plugin to evaluate node centrality;
2. Hub identification using Cytohuba with MCC, DMNC, MNC, and EPC algorithms;
3. Functional clustering via MCODE to detect modules. This workflow systematically characterized network architecture and the biological significance of key components.

### 2.7 GO and KEGG analysis

To systematically characterize the functional annotations of key target genes, we performed Gene Ontology (GO) and Kyoto Encyclopedia of Genes and Genomes (KEGG) pathway enrichment analyses employing a comprehensive bioinformatics workflow. This analysis utilized multiple R packages (version 4.1.2), including org. Hs. eg.db, colorspace, stringi, DOSE, clusterProfiler, pathview, ggplot2, and limma, with a stringent significance threshold of P < 0.05. The GO enrichment analysis was conducted across three ontological domains: Biological Process (BP), Cellular Component (CC), and Molecular Function (MF). For result visualization and interpretation, we leveraged the Metware Cloud platform (https://cloud.metware.cn/) to generate publication-ready graphics. Notably, the top 10 most significantly enriched terms (P < 0.05) within each GO category and KEGG pathway were prioritized for detailed representation, facilitating biological insight extraction.

### 2.8 Molecular docking

Active ingredients from *Fructus Xanthii* were selected and subjected to molecular docking against the predicted core targets. The three-dimensional (3D) structures of these bioactive compounds were retrieved from the PubChem database (https://pubchem.ncbi.nlm.nih.gov), while the structural data for target proteins were obtained from the Protein Data Bank (PDB, https://www.rcsb.org). Molecular docking simulations and visual analysis were carried out using AutoDock Tools 1.5.7 and PyMOL molecular visualization software (Huang et al., 2024b; Huang et al., 2024b). To assess the stability of protein-ligand interactions, molecular dynamics (MD) simulations were applied to the screened potential targets in complex with carboxyatractyloside. Molecular dynamics were performed in GROMACS 2023.2 with CHARMM36/TIP3P; triplicate 100-ns runs per complex. The resulting MD trajectory data were visualized through Xmgrace software version 5.1.25.

### 2.9 Machine learning screening hub genes

We further developed a machine learning model based on the intersection gene expression matrix in the GSE14787 dataset using “caret” (R 4.1.2), encompassing Random Forest (RF), Support Vector Machine (SVM), Generalized Linear Model (GLM), and Extreme Gradient Boosting (XGB). Subsequent screening of intersecting target genes was performed to identify potential hub genes for the treatment of asthma with *Fructus Xanthii*. Models were trained with 10-fold cross-validation; feature importance was summarized by mean decrease accuracy (RF) and SHAP values (XGBoost). GLM residuals were higher, consistent with linear model limitations in small-sample, nonlinear settings. The validity of the models was assessed through residual analysis and ROC evaluation, resulting in the identification of the top 10 variables as hub genes.

### 2.10 Immune infiltration analysis

To investigate potential links between key hub genes and immune microenvironment dynamics in asthma, we utilized the CIBERSORT tool (https://cibersortx.stanford.edu/) for immunophenotyping of 22 cellular subsets within GSE14787 asthma samples. Subsequent application of the ESTIMATE algorithm enabled quantification of tissue immune infiltration characteristics, while Spearman rank correlation analysis systematically assessed gene-immune cell association patterns.

### 2.11 Animal experiment

This study utilized 4- to 6-week-old specific pathogen-free (SPF) female BALB/c mice (weight range: 16–18 g), sourced from Beijing Vital River Laboratory Animal Technology Co., Ltd., with eight mice per experimental group. The asthma model was induced using ovalbumen (OVA, Cat: A5503, Sigma-Aldrich) as the allergen. Following a 3-day acclimatization period, mice were randomly assigned to the following groups: Control group, Model group, Dexamethasone treatment group (1 mg/kg), and *Fructus Xanthii* aqueous extract treatment groups at low (10 mg/kg), medium (20 mg/kg), and high (30 mg/kg) doses. Sensitization was performed on days 0 and 7 by intraperitoneal (i.p.) injection of 20 μg OVA emulsified in 100 μL of 4% aluminum hydroxide adjuvant (Cat: BS010, ZHHC, China) diluted in 100 μL of sterile physiological saline. From days 15–19, mice received daily oral administration of *Fructus Xanthii* aqueous extract or dexamethasone, administered 2 h prior to allergen challenge. Subsequently, for five consecutive days (days 15–19), mice were subjected to 30-min nebulization with 5% OVA to provoke airway inflammation, as outlined in Figure 8A. After the model was established, the mice were anesthetized by intraperitoneal injection of tribromoethanol (0.2 mL/10 g) to facilitate tissue collection. Blood samples were collected via cardiac puncture, and serum was isolated by centrifugation at 850 × g for 10 min at 4 °C, while lung tissues were snap-frozen and stored at −80 °C for subsequent analyses. Bronchoalveolar lavage fluid (BALF) was harvested by exposing the trachea of each mouse, inserting a venous catheter into the cervical trachea, and securing it with sutures. The lungs were lavaged three times with 0.4 mL, 0.5 mL, and 0.6 mL of phosphate-buffered saline (PBS), respectively, and the instilled fluid was gently aspirated using a syringe for downstream immunological and biochemical assessments. Followed by cervical dislocation to confirm death. This method ensures rapid and humane euthanasia, minimizing animal distress. All procedures adhered to ethical guidelines for animal experimentation, ensuring minimal distress and compliance with institutional protocols. All experimental procedures were strictly carried out by the guiding principles of the Animal Care and Use Committee of Qilu Medical University, and the experimental plans were approved by the Laboratory Animal Ethics Committee of Qilu Medical University (YXLL2025R032).

### 2.12 Preparation of *Fructus Xanthii*

Commercial *Fructus Xanthii* was purchased from Omniherb (Daegu, Korea). The material (voucher specimen No. 21032) was authenticated according to the Korean Ministry of Food and Drug Safety (KFDA) guidelines. *Fructus Xanthii* was extracted using hot water to prepare the aqueous extract. The aqueous extract was prepared using a standardized hot water extraction method (1:10 w/v, 100 °C, 2 h × 2), where the yield was approximately 18.5%. The HPLC fingerprint is consistent with published profiles of Fructus Xanthii aqueous extracts, showing major peaks corresponding to xanthatin and phenolic acids (Luo et al., 2021; Han et al., 2022).

### 2.13 qPCR

Primers for mouse *Hsp90ab1, CCNB1, CASP9, PI3K, AKT1,* and the reference gene *GAPDH* were designed using Primer-BLAST (NCBI, https://www.ncbi.nlm.nih.gov/tools/primer-blast/). The primer sequences were: Hsp90ab1-F: 5′- GCT​GAA​GAT​GAC​CGA​CTA​TGA​C-3′, Hsp90ab1-R: 5′- AGT​CCA​GCT​CCA​TGA​TGT​TGA​C-3′; AKT1-F: 5′-ATG​GAC​TAC​CTG​CAC​TCG​GA-3′, AKT1-R: 5′-GAT​GAC​CGT​GTA​GCC​ATT​GT-3′; GAPDH-F: 5′-AGG​TCG​GTG​TGA​ACG​GAT​TTG-3′, GAPDH-R: 5′-TGT​AGA​CCA​TGT​AGT​TGA​GGT​CA-3′; CCNB1-F: 5′- AGA​GGT​GGA​ACT​TGC​TGA​GCC​T-3′, CCNB1-R: 5′- GCA​CAT​CCA​GAT​GTT​TCC​ATC​GG-3′; CASP9-F: 5′- GCT​GTG​TCA​AGT​TTG​CCT​ACC​C-3′, CASP9-R: 5′- CCA​GAA​TGC​CAT​CCA​AGG​TCT​C-3′; PI3k-F: 5′- CTC​TCC​TGT​GCT​GGC​TAC​TGT-3′, PI3K-R: 5′- GCT​CTC​GGT​TGA​TTC​CAA​ACT-3′. Primer specificity was validated by BLAST analysis. Each 20 µL reaction contained 10 µL SYBR Green Master Mix, 0.4 µL forward primer (10 µM), 0.4 µL reverse primer (10 µM), 2 µL cDNA, and 7.2 µL nuclease-free water. Cycling conditions were: initial denaturation at 95 °C for 10 min, followed by 40 cycles of 95 °C for 15 s and 60 °C for 1 min, with a melt curve stage (95 °C for 15 s, 60 °C for 1 min, 95 °C for 15 s) to verify amplicon specificity. Samples were run in triplicate, with no-template controls (NTCs) to detect contamination. *GAPDH* was used for normalization, and relative gene expression was calculated using the 2^−ΔΔCt^ method (ΔCt = Ct (target gene) - Ct (GAPDH), ΔΔCt = ΔCt (sample) - ΔCt (control)). Amplification efficiency was validated by standard curve analysis (95%–105%, *R* ^2^ > 0.99). Data were analyzed using GraphPad Prism 9.0, expressed as mean ± standard deviation (SD).

## 3 Results

### 3.1 Acquisition of the disease targets

**FIGURE 1:**
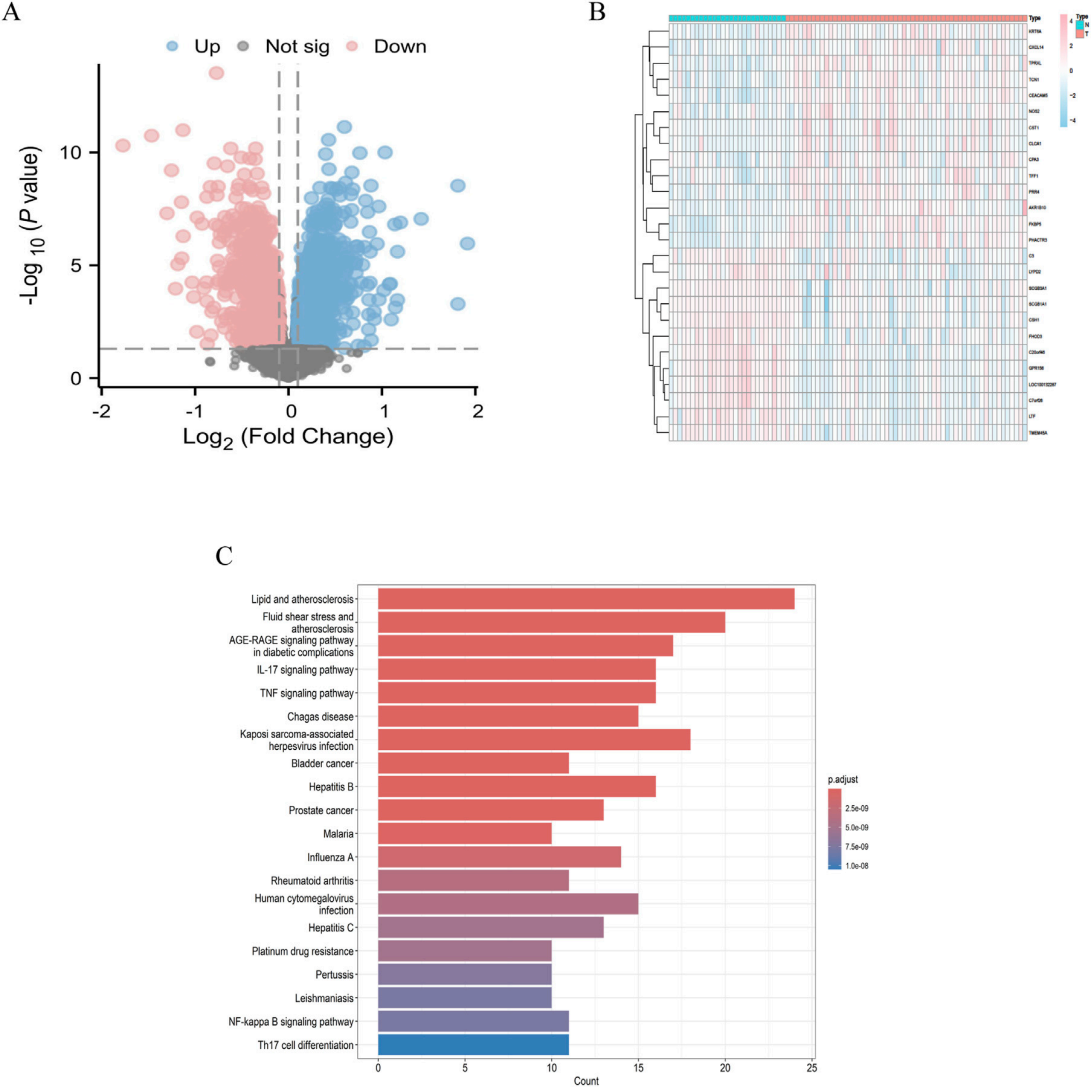
Differential genes in asthma **(A)** Volcano plot depicting differentially expressed genes in asthma; **(B)** Heatmap visualization of differentially expressed genes in asthma; **(C)** Enrichment analysis of signaling pathways for differences.

We downloaded the GSE63142 dataset from the GEO database and conducted data organization and analysis using the R language. A total of 3,755 differentially expressed genes were identified under the criteria of log_2_|FC| ≥ 1 and P < 0.05. Based on these screening results, we generated a volcano plot (Figure 1A) illustrating the differentially expressed genes in asthma. Additionally, we selected upregulated and downregulated genes to create a heatmap (Figure 1B). Among the differentially expressed genes, 1946 were upregulated, including TFF1, FKBP5, AKR1B10, CXCL14, CST1, TPRXL, NOS2, CPA3, KRT6A, PHACTR3, TCN1, CLCA1, and CEACAM5, among others. Conversely, 1809 genes were downregulated, including LOC100132287, C7orf26, LTF, GPR156, SCGB3A1, SCGB1A1, CSH1, TMEM45A, C20orf46, FHOD3, C3, LYPD2, among others. These differentially expressed genes are involved in various signaling pathways (Figure 1C).

### 3.2 Asthma-related core gene clusters

**FIGURE 2:**
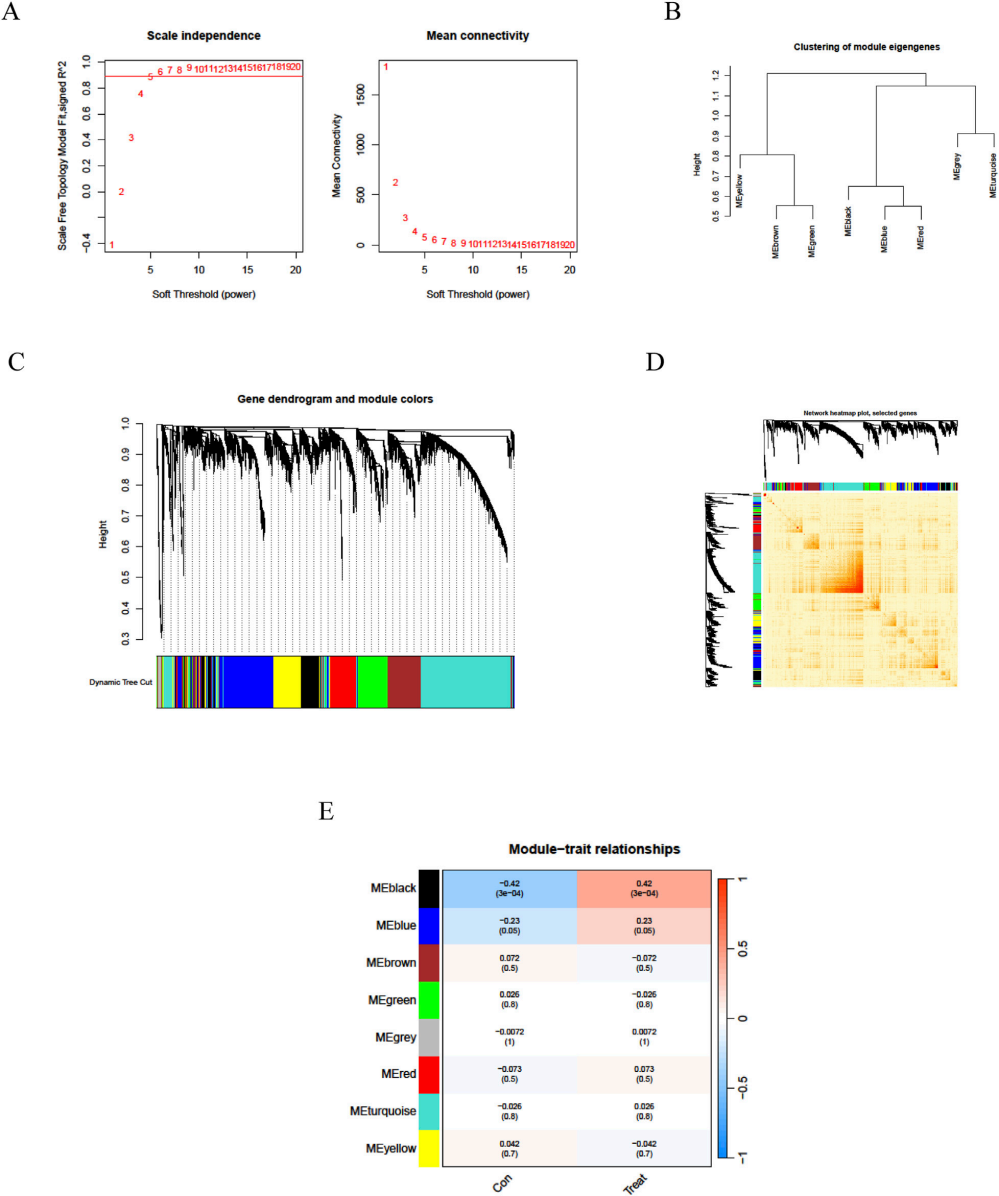
Weighted gene co-expression network analysis of asthma. **(A)** WGCNA soft threshold power selection plot; **(B)** Gene dendrogram based on WGCNA clustering; **(C)** Topological Overlap Matrix (TOM) visualization; **(D)** WGCNA Gene heatmap. **(E)** Heatmap depicting module-trait relationships with associated correlation values and significance levels (red indicates positive correlation, blue indicates negative correlation; values in cells are correlation coefficients with p-values in parentheses).

We employed the Weighted Gene Co-expression Network Analysis (WGCNA) methodology to analyze the GSE14787 dataset and construct a gene co-expression network for identifying key gene modules associated with asthma. Initially, we detected and eliminated outliers from abnormal samples. Subsequently, we constructed a scale-free network and utilized the pickSoftThreshold function to select the optimal soft power β = 18 (*R* ^2^ = 0.962) (Figure 2A). We then established an adjacency matrix, converted it into a topological overlap matrix (TOM), and applied varying degrees to generate a gene tree diagram with corresponding module colors (Figures 2B–D). Our analysis revealed that MEblack and MEblue modules exhibited significant positive correlations with asthma samples, with correlation coefficients of 0.42 and 0.23, respectively. Consequently, the MEblack gene cluster was selected for further investigation (Figure 2E). Genes within each module were filtered based on their significance and module correlation, with thresholds set at geneSigFilter = 0.5 and moduleSigFilter = 0.5. The results identified MEblack as a cluster containing 741 asthma-related core genes, including POMP, GUSBL2, RBM39, PSMA6, RFX1, TCEB1, PSMD6, and others.

### 3.3 Target prediction of *Fructus Xanthii* in the treatment of asthma

**FIGURE 3:**
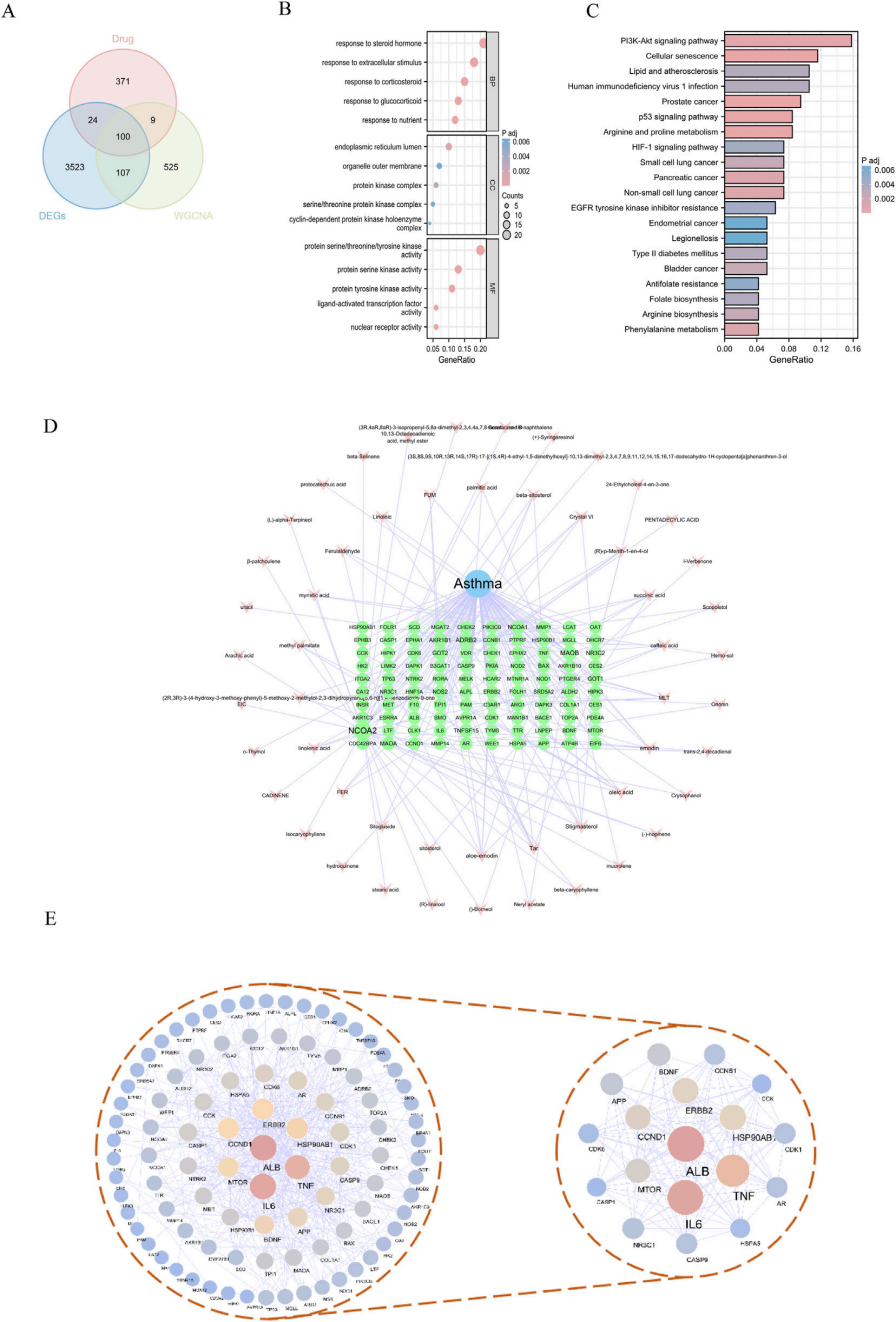
Target Prediction of *Fructus Xanthii* in the treatment of asthma **(A)** Venn diagram illustrating the intersection targets of *Fructus Xanthii* in asthma treatment; **(B)** GO enrichment analysis therapeutic effects of *Fructus Xanthii* on asthma; **(C)** KEGG pathway enrichment analysis of *Fructus Xanthii* in asthma treatment; **(D)** Network visualization of disease-drug-active ingredient-target interactions; **(E)** Protein-protein interaction network revealing therapeutic mechanisms of *Fructus Xanthii* in asthma.

Through the application of TCMSP and subsequent searches in the SwissTarget Prediction database, we identified the chemical composition and potential targets of *Fructus Xanthii*, yielding a total of 1,317 potential targets. By intersecting these targets with 741 asthma gene clusters and 3,755 asthma DEGs, we identified 100 specific targets through which *Fructus Xanthii* may exert therapeutic effects on asthma (Figure 3A).

To elucidate the functional roles of the 100 overlapping genes, a comprehensive functional enrichment analysis was performed. Gene Ontology (GO) annotation revealed 1,275 significantly enriched terms across three ontological categories—biological processes, cellular components, and molecular functions—in the context of *Fructus Xanthii*’s therapeutic intervention in asthma. Biological process terms dominated the enrichment landscape (1,153 entries), with prominent clusters including steroid hormone response, glucocorticoid/corticosteroid responsiveness, nutrient/extracellular stimulus adaptation, and related signaling cascades. Cellular component annotations (31 entries) highlighted endoplasmic reticulum subcompartments, protein kinase complexes (e.g., cyclin-dependent holoenzymes, serine/threonine kinase complexes), and organelle membrane structures. Molecular function enrichment (91 entries) prioritized kinase activities (serine/threonine/tyrosine-specific), nuclear receptor signaling, and ligand-dependent transcription factor regulation (Figure 3B).

KEGG functional enrichment analysis identified 102 related pathways involved in the intervention of *Fructus Xanthii* in asthma, mainly including Arginine and proline metabolism, Prostate cancer, Cellular senescence, p53 signaling pathway, and PI3K-Akt signaling pathway (Figure 3C). The intersecting targets were imported into Cytoscape 3.8.1 software to construct a “disease-drug-active ingredient-target” network (Figure 3D). Additionally, a protein interaction network was constructed using the intersecting genes, with nodes assigned blue-yellow-wine red gradients based on degree values. The network analysis revealed 96 nodes with 503 interrelationships. The highest-ranked targets in terms of degree were ALB, IL6, TNF, HSP90AB1, ERBB2, and CCND1, among others (Table 1). By screening for core targets with degree values greater than twice the median, a network comprising 18 nodes and 120 interrelationships was identified (Figure 3E).

**TABLE 1:** Topological data of protein interaction network (Partial).

Affinity (kcal/mol)	CASP9	CCK	CCNB1	CDK6	ERBB2	HSP90AB1	NR3C1
24-Ethylcholest-4-en-3-one	−7.91	−5.35	−8.04	−8.11	−8.04	−8.73	−7.09
Aloe-emodin	−5.81	−4.5	−6.27	−6.17	−5.57	−6.31	−5.91
Beta-sitosterol	−7.68	−5.26	−8.12	−7.82	−7.34	−8.79	−7.58
Carboxyatractyloside	−9.89	−6.8	−10.04	−9.36	−9.35	−10.09	−9.14
CID_442510	−7.39	−5.34	−7.13	−7.26	−7.4	−8.22	−7.41
CID_6916037	−7.68	−5.7	−8.11	−8.19	−7.6	−8.73	−7.95
Cynarin(e)	−8.74	−6.36	−8.43	−8.02	−7.7	−9.23	−9.28
Moupinamide	−6.66	−5.48	−6.79	−6.82	−6.54	−7.59	−7.61
Peroxyergostero	−7.97	−5.3	−8.56	−7.54	−7.85	−9.19	−7.48
Sitosterol	−7.52	−5.3	−8.09	−8.06	−7.5	−8.8	−7.8
Stigmasterol	−7.87	−5.11	−7.91	−7.29	−7.84	−8.7	−7.9

### 3.4 Identification of target hub genes with machine learning

**FIGURE 4:**
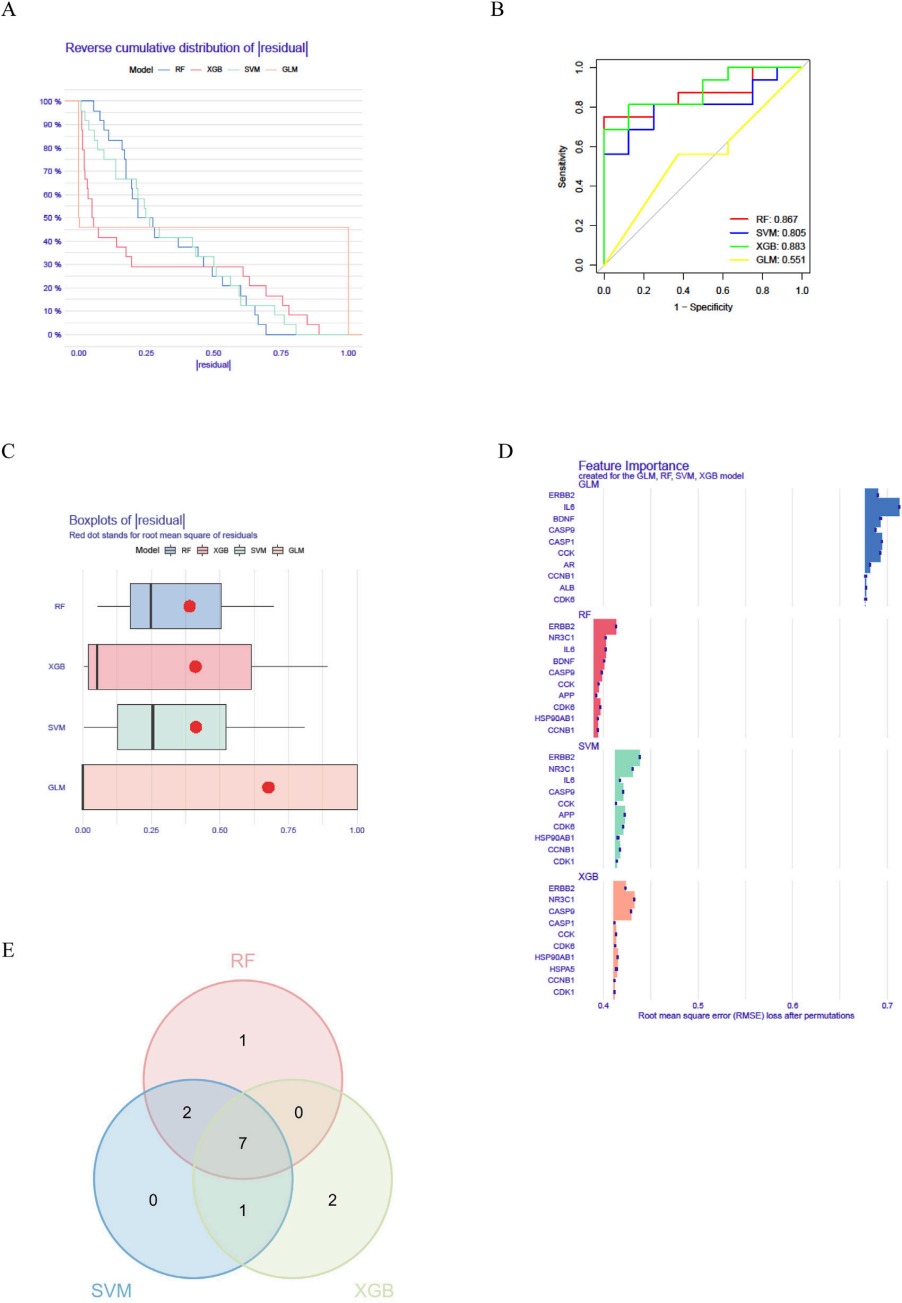
Identification of key genes involved in the treatment of asthma with *Fructus Xanthii* **(A)** Machine learning residual evaluation; **(B)** Machine learning residual evaluation; **(C)** Machine learning ROC evaluation; **(D)** Identification of key genes; **(E)** Three intersections of machine learning.

To further investigate the core genes within the network, we identified a network comprising 16 targets as the core by applying a threshold of degree values exceeding twice the median. We developed a machine learning model using the “caret” package in R and assessed its validity through residual analysis and ROC evaluation. The results demonstrated that the Random Forest (RF), Support Vector Machine (SVM), and XGBoost (XGB) machine learning methods yielded small residuals, whereas the Generalized Linear Model (GLM) produced larger residuals (Figures 4A,B). Concurrently, ROC evaluation revealed that RF, SVM, and XGB methods all achieved values greater than 0.7, with XGBoost exhibiting the highest ROC value of 0.883 (Figure 4C). Based on a comprehensive analysis of both residuals and ROC performance, we selected the RF, SVM, and XGB models for subsequent analysis and identified the top 10 variables from each model as central genes (Figure 4D). The intersection of these three machine learning approaches revealed 7 core targets (Figure 4E): HSP90AB1, CCNB1, CCK, CDK6, CASP9, NR3C1, and ERBB2.

### 3.5 Molecular docking virtual verification

**FIGURE 5:**
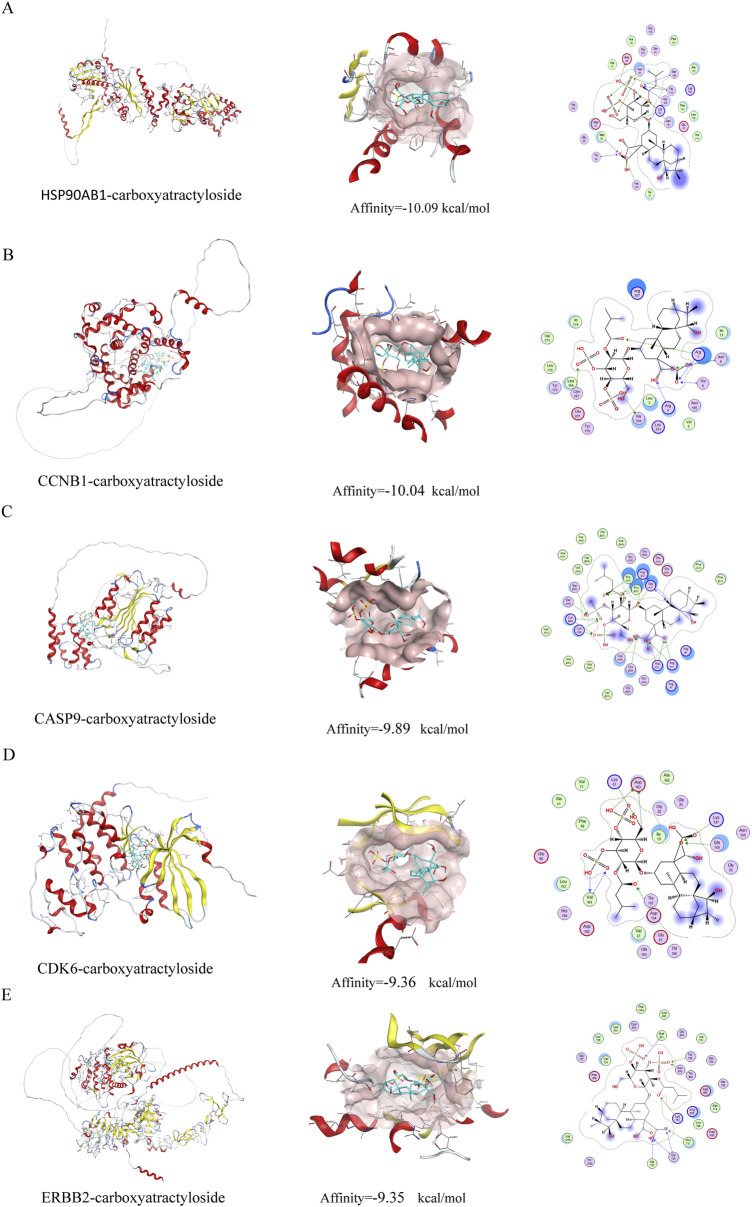
Schematic diagram of molecular docking. **(A)** HSP90AB1-carboxyatractyloside; **(B)** CCNB1-carboxyatractyloside; **(C)** CASP9-carboxyatractyloside; **(D)** CDK6-carboxyatractyloside; **(E)** ERBB2-carboxyatractyloside.

Molecular docking analysis was conducted to evaluate the binding affinities of eleven compounds against seven key target genes (HSP90AB1, CCNB1, CCK, CDK6, CASP9, NR3C1, ERBB2), with interaction scores ranging from −5.3 kcal/mol to −10.09 kcal/mol (see Table 1). A lower docking score correlates with a stronger binding affinity, with scores below −5.0 kcal/mol indicating potential interactions and those below −7.0 kcal/mol suggesting robust binding affinities. Among the targets, Carboxyatractyloside demonstrated the highest binding affinity for HSP90AB1. The binding interactions of the five key targets with their respective ligands are shown in Figure 5. The binding pockets of HSP90AB1, CCNB1, CDK6, CASP9, and ERBB2 were effectively occupied by the selected ligands, with stability provided through hydrogen bonds and hydrophobic interactions. In particular, Carboxyatractyloside formed hydrogen bonds with LYS53 of HSP90AB1 at a distance of 3.00 Å (see Figure 5A). The binding sites and affinities for the other proteins and ligands are presented in Figures 5B–E. These results highlight the effective binding of the five compounds to the core targets through hydrogen bonds and hydrophobic interactions.

### 3.6 Molecular dynamics simulations

**FIGURE 6:**
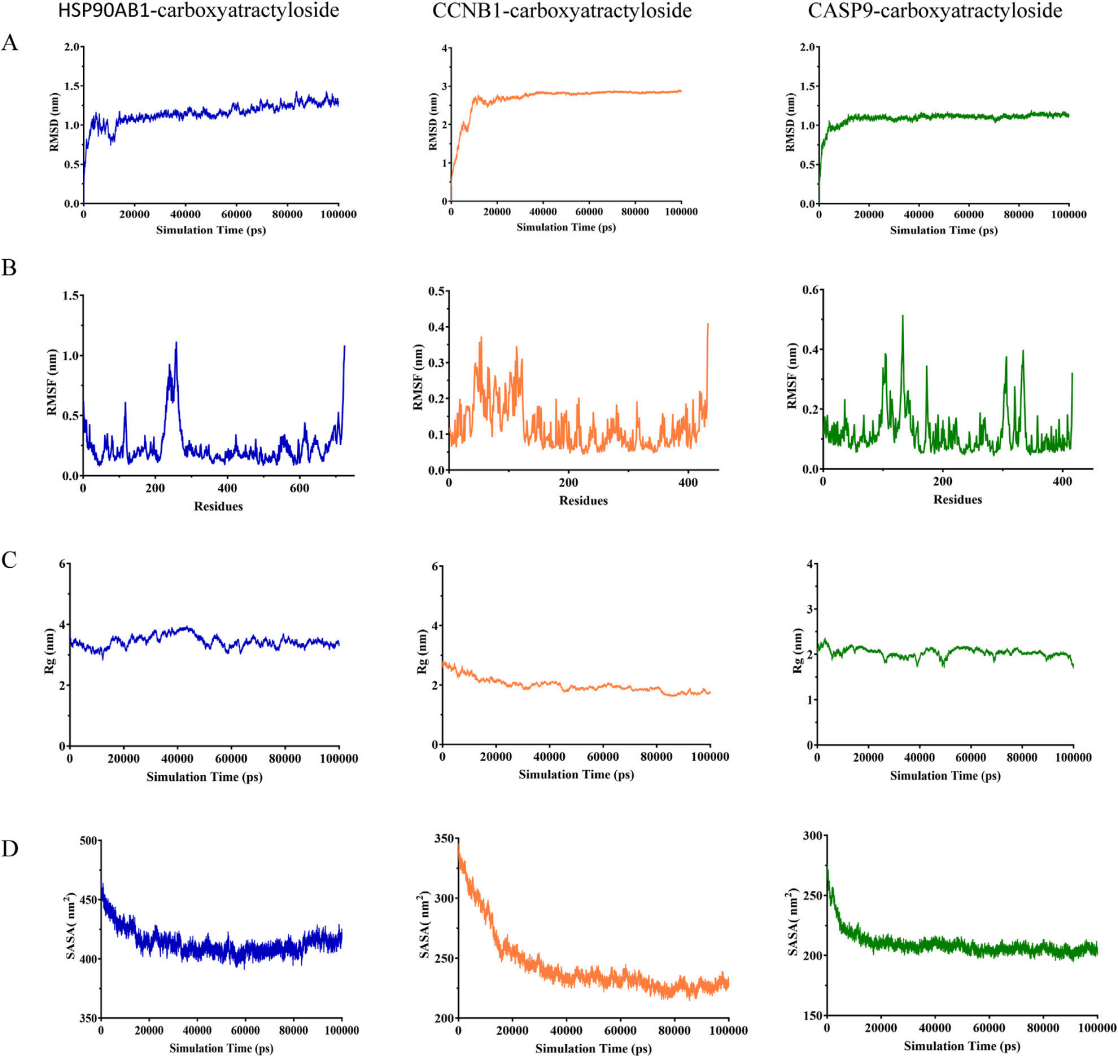
Molecular dynamics simulation and binding free energy calculation **(A)** RMSD values of the three complexes. **(B)** RMSF values of the three complexes. **(C)** Radius of gyration (Rg) values of the three complexes. **(D)** Solvent-accessible surface area (SASA) values of the three complexes.

To characterize the dynamic stability of the top 3 protein-ligand complexes (HSP90AB1/CCNB1/CASP9-Carboxyatractyloside), we performed 100-ns MD simulations with triplicate sampling. Structural equilibration was verified through root mean square deviation (RMSD) trajectories, where values below 3 nm (Figure 6A) confirmed physiological stability across all systems. Post-equilibration RMSD plateaued at 1.16 ± 0.13 Å (HSP90AB1) to 2.85 ± 0.18 Å (CCNB1), indicating ligand-specific stabilization patterns. Atomic positional fluctuations were quantified via root mean square fluctuation (RMSF) analysis (Figure 6B), revealing constrained molecular motions with values ranging from 0.19 ± 0.06 Å (CCNB1) to 0.47 ± 0.23 Å (HSP90AB1). Compactness metrics derived from radius of gyration (Rg) calculations (Figure 6C) showed sustained structural integrity throughout simulations (2.18 ± 0.11 nm [CCNB1] - 3.26 ± 0.15 nm [HSP90AB1]). The SASA values of the three complexes also demonstrated stability, reaching average values of 420.87 ± 3.79 nm^2^, 241.87 ± 8.62 nm^2^, and 215.76 ± 3.49 nm^2^, respectively.

### 3.7 Immune infiltration analysis

**FIGURE 7:**
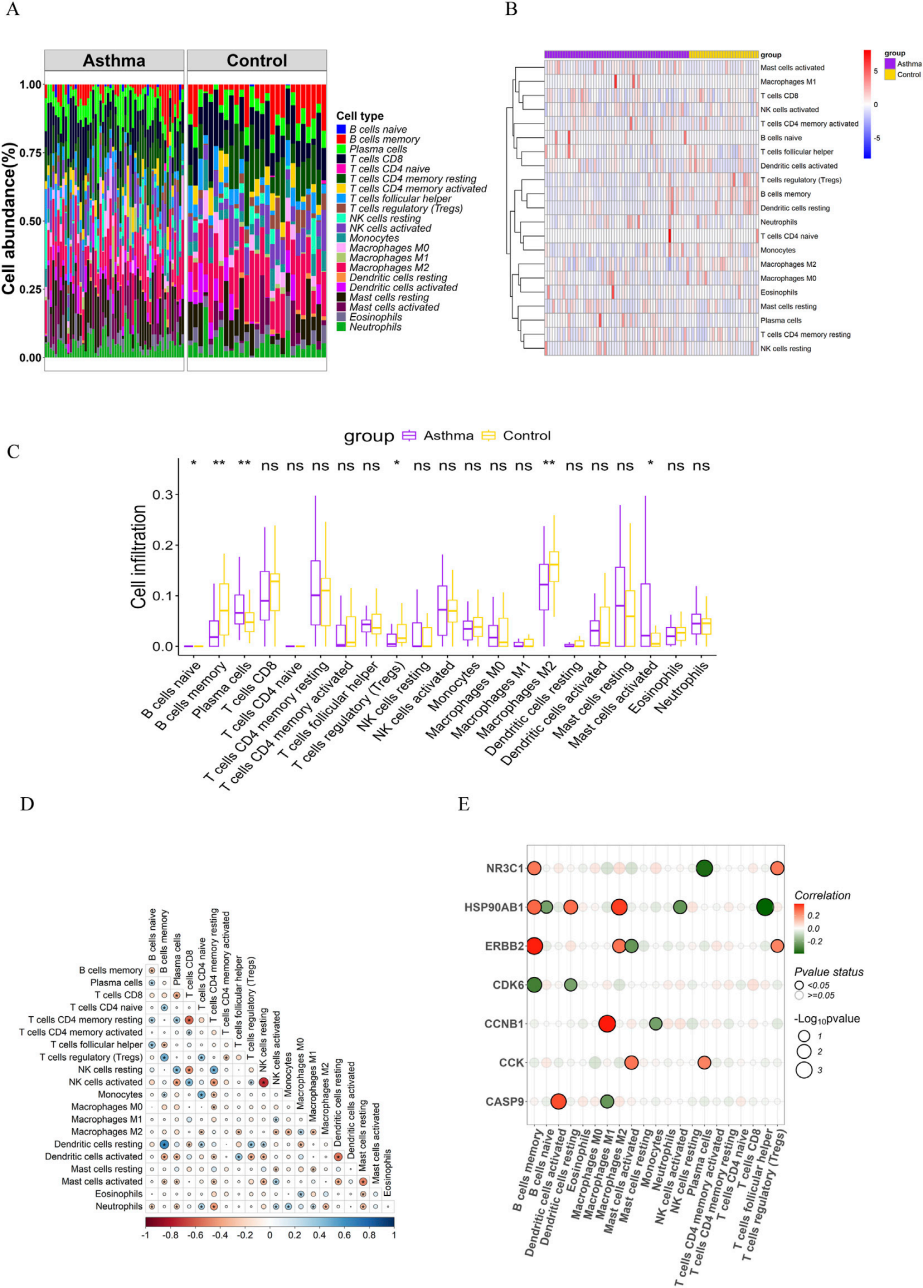
Immune infiltration analysis of target hub genes **(A)** Proportional histograms depicting the distribution of 22 immune cell types; **(B)** Heatmap illustrating the expression abundance of 22 immune cell populations; **(C)** Box plots showing differential expression of 22 immune cell types between normal and DN groups; **(D)** Correlation heatmap of immune cell populations. **(E)** Heat map of the correlation between immune infiltration and the hub genes’ expression.

We initially employed the CIBERSORT algorithm to characterize immune cell composition across 22 distinct populations in GSE14787 samples (Figures 5A,B). Comparative analysis between asthmatic and non-asthmatic tissues revealed significant alterations in immune cell profiles (Figure 5C). The findings revealed (Figures 7C,D) that M2 macrophages and memory B cells were significantly decreased in asthma samples, whereas plasma cells were significantly elevated, suggesting the importance of focusing on macrophages, plasma cells, and memory B cells in future investigations. To explore functional relationships, we subsequently investigated correlations between the expression of key hub genes and immune cell infiltration profiles (Figure 5D), providing insights into potential regulatory networks linking gene expression to immune microenvironment composition. The heatmap reveals distinct immunomodulatory roles for core asthma targets: NR3C1 exhibits strong positive correlations with regulatory immune cells—notably memory B cells (r ≈ 0.64) and T follicular helper cells (r ≈ 0.48)—suggesting its role in enhancing immunosuppressive responses, consistent with its function as the glucocorticoid receptor. Conversely, HSP90AB1 shows divergent associations: positive with pro-inflammatory M1 macrophages (r ≈ 0.38) but negative with anti-inflammatory M2 macrophages (r ˜ −0.41), indicating its involvement in macrophage polarization imbalance. ERBB2 displays broad negative correlations with lymphocyte populations, including memory B cells (r ˜ −0.71) and plasma cells (r ˜ −0.43), aligning with its known role in mucus hypersecretion and adaptive immune suppression. Critically, NR3C1, HSP90AB1, and ERBB2 collectively suppress plasma cells (unified negative correlations), implicating synergistic control of humoral immunity dysregulation in asthma. CDK6 and CCNB1 show moderate negative associations with neutrophils and mast cells, while CCK exhibits no significant immune correlations (p > 0.05), implying non-immune mechanistic pathways.

### 3.8 *Fructus Xanthii* aqueous extract enhanced pulmonary function and mitigated inflammatory infiltration of type II immune response in an OVA-Induced murine asthma model

**FIGURE 8:**
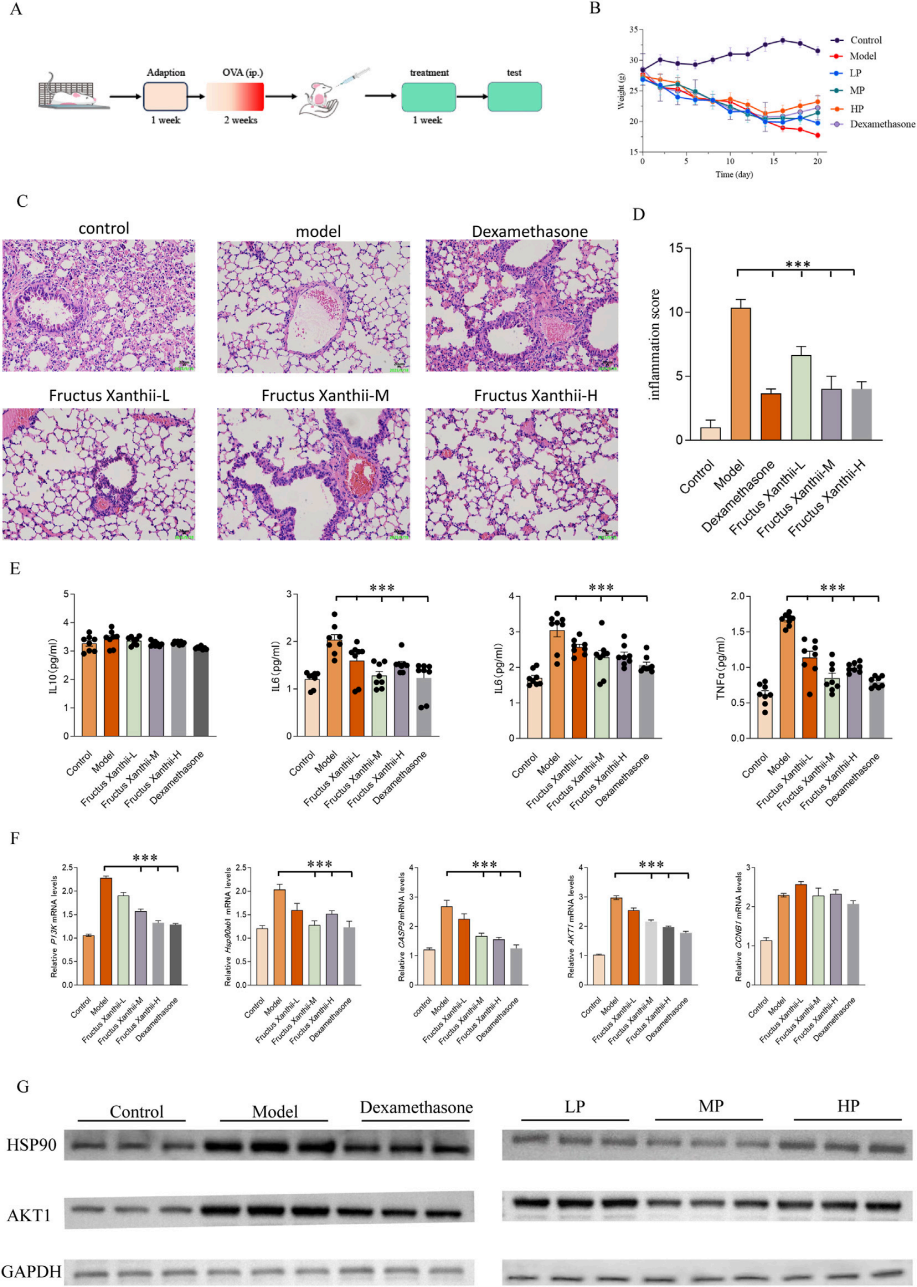
Comprehensively evaluates the therapeutic potential of *Fructus Xanthii* aqueous extract in an ovalbumin (OVA)-induced allergic asthma murine model, detailing the experimental timeline **(A)**, body weight dynamics **(B)**, histopathological lung alterations via H&E staining **(C)**, inflammation scores **(D)**, cytokine profiles in bronchoalveolar lavage fluid (BALF) via ELISA **(E)**, and mRNA expression of key genes (Hsp90ab1, CCNB1, CASP9, PI3K, AKT1) via qPCR **(F)** in BALB/c mice. The protocol involved a 1-week adaptation, 2-week OVA sensitization, and 1-week treatment with low (L), medium (M), or high (H) doses of *Fructus Xanthii* extract or dexamethasone, followed by multifaceted assessments. Body weight declined in the Model group but was mitigated dose-dependently by *Fructus Xanthii* and dexamethasone **(B)**. Histopathology revealed severe inflammation in the Model group, with progressive amelioration in treated groups, particularly at higher *Fructus Xanthii* doses and dexamethasone **(C)**, corroborated by reduced inflammation scores **(D)**. Cytokine levels (TNF-α, IL-6, IL-1β, IL-5) were significantly elevated in the Model group, with notable suppression by *Fructus Xanthii* and dexamethasone **(E)**. Gene expression analysis indicated upregulated Hsp90ab1, CCNB1, CASP9, PI3K, and AKT1 in the Model group, with dose-dependent downregulation by treatments, suggesting modulation of stress response, cell cycle, apoptosis, and survival pathways **(F)**. Data, presented as mean ± SEM (n = 8), highlight *Fructus Xanthii*’s concentration-dependent anti-inflammatory and anti-asthmatic efficacy, comparable to dexamethasone, with statistical significance (***p < 0.001) established via ANOVA with Tukey’s test. **(G)** Western blot analysis of HSP90AB1 and AKT1 protein levels in lung tissues, with GAPDH as the loading control, showing increased expression in the Model group and normalization in treated groups. All data are expressed as mean ± SEM (n = 8 per group); statistical significance was assessed by one-way ANOVA followed by Tukey’s multiple comparisons test.

Figure 8 provides a comprehensive evaluation of the therapeutic efficacy of *Fructus Xanthii* aqueous extract in mitigating ovalbumen (OVA)-induced allergic asthma in a murine model, integrating the experimental design, physiological responses, histopathological observations, inflammatory metrics, cytokine profiles, and molecular gene expression analyses in BALB/c mice subjected to OVA sensitization and challenge. The study protocol (A) outlines a 1-week adaptation period, followed by a 2-week OVA sensitization phase via intraperitoneal (i.p.) injection to establish the asthma model, and a subsequent 1-week treatment phase where groups received either vehicle (for control and model groups), low (L), medium (M), or high (H) concentrations of *Fructus Xanthii* aqueous extract, or dexamethasone as a positive control drug. Final assessments included body weight monitoring, bronchoalveolar lavage fluid (BALF) collection, histopathological examination using hematoxylin and eosin (H&E) staining, inflammation scoring, enzyme-linked immunosorbent assay (ELISA) for cytokine quantification, and real-time quantitative polymerase chain reaction (qPCR) for gene expression profiling. Body weight dynamics (B) reveal a significant time-dependent decline in the Model group, reflecting systemic inflammation or anaphylactic stress induced by OVA, which was progressively alleviated by *Fructus Xanthii* extract in a dose-dependent manner, with high concentrations nearing the protective effect of dexamethasone, suggesting potential anti-cachectic or immunomodulatory benefits. Histopathological analysis (C) showcases pristine alveolar structures in the Control group, contrasted by severe asthmatic features in the Model group—such as thickened alveolar septa, dense peribronchial and perivascular leukocyte infiltration (predominantly eosinophils, lymphocytes, and macrophages), bronchial epithelial hypertrophy, goblet cell metaplasia with excessive mucus, and luminal narrowing—while dexamethasone and *Fructus Xanthii* extract treatments, particularly at medium and high doses, restored alveolar integrity and reduced inflammatory cell aggregation in a concentration-dependent manner, indicating glucocorticoid-like anti-inflammatory activity (scale bars: 100 μm upper panels, 50 μm lower panels). Inflammation scores (D) derived from H&E histopathology highlight a marked elevation in the Model group (***p < 0.001 *versus* Control), indicative of robust Th2-driven airway inflammation, which was significantly attenuated by dexamethasone and higher doses of *Fructus Xanthii* extract (**p < 0.01 or *p < 0.05 *versus* Model), underscoring effective suppression of inflammatory cascades (mean ± SEM, n = 8). Cytokine quantification in BALF (E) demonstrates substantial increases in tumor necrosis factor-alpha (TNF-α), interleukin-6 (IL-6), interleukin-1 beta (IL-1β), and interleukin-5 (IL-5) in the Model group (***p < 0.001 *versus* Control), reflecting enhanced eosinophilic recruitment, innate immune activation, and pleiotropic inflammation, which were dose-dependently reduced by *Fructus Xanthii* extract, with high-dose effects matching dexamethasone’s potent inhibition (**p < 0.01 or *p < 0.05 *versus* Model), suggesting a therapeutic modulation of cytokine networks. Molecular analysis via qPCR (F) reveals significant upregulation of heat shock protein 90ab1 (Hsp90ab1), cyclin B1 (CCNB1), caspase-9 (CASP9), phosphoinositide 3-kinase (PI3K), and protein kinase B isoform 1 (AKT1) in the Model group (***p < 0.001 *versus* Control), implicating heightened chaperone activity, cell cycle progression, apoptosis, and PI3K/AKT-mediated survival signaling in asthma-aggravated lung injury; these were dose-dependently downregulated by *Fructus Xanthii* extract, with high-dose effects paralleling dexamethasone (**p < 0.01 or *p < 0.05 *versus* Model), potentially through interference with stress responses, cell cycle arrest, apoptosis induction, or PI3K/AKT inhibition to limit inflammatory hyperplasia. All data are presented as mean ± SEM (n = 8), with statistical significance assessed by one-way ANOVA followed by Tukey’s multiple comparisons test, collectively affirming the concentration-dependent anti-inflammatory and anti-asthmatic properties of *Fructus Xanthii* aqueous extract, comparable to dexamethasone. Western blot analysis further validated these findings at the protein level, showing increased expression of HSP90AB1 and AKT1 in the Model group, with normalization in treated groups, using GAPDH as the loading control (Figure 8G). All data are presented as mean ± SEM, with statistical significance determined by one-way ANOVA followed by Tukey’s multiple comparisons test. These results collectively demonstrate the concentration-dependent anti-inflammatory and anti-asthmatic effects of *Fructus Xanthii* aqueous extract, comparable to dexamethasone, through modulation of inflammatory cytokines and key signaling pathways.

## 4 Discussion

Our integrated analysis demonstrates that asthma involves complex transcriptional dysregulation, as evidenced by the identification of 3,755 differentially expressed genes (DEGs). Specifically, WGCNA highlighted the MEblack module (741 genes), enriched in proteostasis regulators (e.g., POMP, PSMA6) and immune signaling components, as a key driver of asthma pathogenesis (Dong et al., 2017). This suggests a dual-pronged disease mechanism involving both immune activation and impaired epithelial stress responses, a paradigm that extends beyond traditional inflammatory models. By integrating *Fructus Xanthii*’s 1,317 predicted targets with the asthma-related genes, we identified 100 overlapping targets, which were significantly enriched in pathways related to steroid hormone response (e.g., glucocorticoid receptor NR3C1), kinase activity (e.g., HSP90AB1), and immune modulation. Crucially, KEGG analysis revealed the PI3K-AKT and p53 signaling pathways, central regulators of airway remodeling and cellular senescence, respectively, as key targets of *Fructus Xanthii*. Further refinement using machine learning (RF/SVM/XGBoost) pinpointed seven hub genes: HSP90AB1, CCNB1, CASP9, CDK6, NR3C1, ERBB2, and CCK. Of particular interest is the HSP90AB1-NR3C1 axis. Molecular docking revealed that Carboxyatractyloside, a key compound in *Fructus Xanthii*, binds with high affinity (−10.09 kcal/mol) to LYS53 of HSP90AB1, potentially stabilizing its ATPase domain and preventing NR3C1 degradation. This mechanism could directly address glucocorticoid resistance, a hallmark of severe asthma. Molecular dynamics simulations confirmed the stable binding (RMSD <3 Å) of Carboxyatractyloside to HSP90AB1, CCNB1, and CASP9, demonstrating compact and stable complex dynamics (Rg: 2.18–3.26 nm). Immune infiltration analysis further supported the relevance of these targets, linking HSP90AB1 and ERBB2 expression to M2 macrophage polarization and highlighting asthma-specific immune dysregulation. The GEO datasets (GSE63142 and GSE14787) predominantly comprised samples from patients with severe asthma (Porsbjerg et al., 2023). While this focused enabled robust identification of disease-associated signals, generalizability to mild or intermittent asthma phenotypes remained uncertain, as these subgroups might exhibit distinct molecular signatures and inflammatory profiles (Kuo CS, et al. J Allergy Clin Immunol 2017; 139:1797–1807). Second, carboxyatractyloside, despite demonstrating the strongest binding affinity to HSP90AB1 (−10.09 kcal/mol), is a known inhibitor of the mitochondrial ADP/ATP translocase and has been associated with hepatotoxicity and nephrotoxicity in preclinical models (Obatomi and Bach, 1998). Its clinical translation would require comprehensive tiered toxicological profiling (acute, sub-chronic, and genotoxicity studies) prior to any therapeutic consideration. Third, the current study relies entirely on computational predictions and *in vivo* observations without *in vitro* mechanistic validation. Functional consequences of modulating hub genes via siRNA-mediated knockdown or lentiviral overexpression in human bronchial epithelial cells or macrophage models have not been evaluated. Such experiments are essential to confirm target engagement, downstream pathway modulation, and phenotypic outcomes. Future studies should include cell-based knockdown/overexpression and systematic toxicology to substantiate translational relevance. *In vivo* experiments in an OVA-sensitized murine model corroborated these findings, demonstrating *Fructus Xanthii* aqueous extract’s dose-dependent efficacy: attenuated body weight loss, restored lung histopathology (reduced peribronchial infiltration, epithelial hypertrophy, and mucus hypersecretion via H&E), lowered inflammation scores, suppressed BALF cytokines (TNF-α, IL-6, IL-1β, IL-5), and downregulated hub gene expression (HSP90AB1, CCNB1, CASP9, PI3K, AKT1), rivaling dexamethasone. This validates *Fructus Xanthii*’s role in blocking NF-κB/MAPK cascades, inhibiting extracellular HSP90α-mediated RhoA/MLC signaling, and preserving epithelial barrier integrity, as extracellular HSP90α exacerbates bronchial dysfunction in asthma.

Acute asthma exacerbations are strongly correlated with inflammatory mediators released by infiltrating immune cells in the airways. These mediators can induce epithelial cell damage and programmed cell death, disrupting airway mucosal integrity and function. Notably, damaged epithelial tissue may further amplify the innate immune response by persistently releasing endogenous danger signals, including damage-associated molecular patterns (DAMPs), which act as triggers for immune activation (Méndez-Enríquez and Hallgren, 2019; Miller et al., 2021). This persistent immune activation triggered by cell damage eventually leads to abnormal airway structure remodeling and promotes the chronic process of the disease. Based on this pathological mechanism, modern asthma treatment strategies should focus on targeting and regulating the key nodes in the inflammatory cascade reaction, while exploring new therapeutic approaches to block the abnormal death pathways of epithelial cells. Hsp90 is compositively expressed in eukaryotes and upregulated under stress. Its subtype Hsp90α (HSP90AB1) is distributed in the cytoplasm and the surface of some cells, and can be actively secreted into the intercellular space, participating in wound healing, inflammation regulation, and cell migration. Airway epithelial barrier injury is associated with asthma. Secretory Hsp90α intensifies the pathological process through pro-inflammatory effects, while Hsp90 inhibitors can effectively alleviate asthma-induced epithelial barrier dysfunction (Ye et al., 2019; Huang et al., 2024a). Extracellular HSP90α (eHSP90α) can activate the RhoA/myosin light chain (MLC) signal transduction pathway, leading to continuous phosphorylation of MLC and cytoskeletal remodeling, disrupting the bronchial epithelial barrier and resulting in the deficiency of two essential adhesion connexin proteins, E-cadherin and β-catenin. The disruption of the integrity of this epithelial barrier can lead to increased permeability of the bronchial epithelium, intensifying the inflammatory response and the development of asthma (Dong et al., 2017). In addition, studies have found that the glucocorticoid receptor (GR) encoded by the NR3C1 gene, as a core regulator of immune and inflammatory response, is a therapeutic target in asthma and other chronic inflammatory diseases. Corticosteroid drugs (such as hydrocortisone, prednisone, etc.) exert anti-inflammatory effects precisely by activating GR (Panek et al., 2015; Pan et al., 2022). The enrichment of NR3C1 in “response to corticosteroid” pathways, downstream of HSP90AB1, suggests that *Fructus Xanthii* may restore glucocorticoid sensitivity by stabilizing GR through HSP90AB1 chaperone function. ERBB2, often overexpressed in asthmatic epithelium, drives mucus hypersecretion. Its inhibition by *Fructus Xanthii* components could further mitigate airway remodeling (Hough et al., 2020).

While our bioinformatics analysis of public datasets provides valuable insights into the potential therapeutic mechanisms of *Fructus Xanthii* in asthma, the computational predictions, including molecular docking and machine learning results, require experimental validation. Future *in vitro* and *in vivo* studies are needed to confirm predicted target interactions (e.g., Carboxyatractyloside binding) and assess pharmacological efficacy in diverse patient populations. Furthermore, rigorous evaluation of Carboxyatractyloside’s bioavailability, potential toxicity (especially hepatotoxicity), and therapeutic efficacy is crucial before clinical translation. Given the complexity of *Fructus Xanthii*’s chemical composition, further studies should focus on isolating and characterizing the specific active constituents. Finally, while our immune infiltration analysis provides valuable insights, it relies on computational deconvolution of bulk RNA-seq data. Single-cell RNA sequencing would provide a more granular understanding of the immune landscape in asthma and the cell-type-specific effects of *Fructus Xanthii*.

Despite these limitations, our study identifies seven core targets (HSP90AB1, CCNB1, CCK, CDK6, CASP9, NR3C1, and ERBB2) that offer promising avenues for developing novel asthma therapies. Through the integration of network pharmacological analysis, machine learning, and molecular dynamics simulation technology, we screened the potential targets of *Fructus Xanthii* for the treatment of asthma. These key molecular targets identified by these screens are potential intervention nodes for core biological processes such as mediating inflammatory cascades, cell cycle regulation, and immune homeostasis maintenance. Further validation experiments targeting these potential targets will be carried out in subsequent studies.

In conclusion, several potential core targets of *Fructus Xanthii* for the treatment of asthma were screened out in this study. They may serve as potential biomarkers for the diagnosis of asthma and the monitoring of disease severity. In addition, targeted therapy against these molecules may also bring more effective treatment strategies to asthma patients. Finally, the differences in different immune cells found in this study between asthma patients and non-asthma patients need further research. By exploring the differential expression of the potential targets of *Fructus Xanthii* in different immune cells, it is helpful to understand the therapeutic potential of *Fructus Xanthii* and provide new ideas for the development of innovative asthma treatment methods.

## Data Availability

The datasets presented in this study can be found in online repositories. The names of the repository/repositories and accession number(s) can be found in the article/supplementary material.
